# Quality and readability of the dental information obtained by patients on Internet: criteria for selecting medical consultation websites

**DOI:** 10.4317/medoral.23521

**Published:** 2020-05-10

**Authors:** Sara Allegue-Magaz, Miguel Peñarrocha-Diago, Cintia M Chamorro-Petronacci, Alejandro I Lorenzo-Pouso, Andrés Blanco-Carrión, Abel García-García, Mario Pérez-Sayáns

**Affiliations:** 1 DDS. Oral Medicine, Oral Surgery and Implantology Unit. Faculty of Medicine and Dentistry. University of Santiago de Compostela; 2 MD, DDS, PhD. Stomatology Department, Faculty of Medicine and Dentistry, University of Valencia, Spain; 3 PhD. Oral Medicine, Oral Surgery and Implantology Unit. Faculty of Medicine and Dentistry. University of Santiago de Compostela; 4 MD, DDS, PhD. Oral Medicine, Oral Surgery and Implantology Unit. Faculty of Medicine and Dentistry. University of Santiago de Compostela; 5 MD, PhD. Oral Medicine, Oral Surgery and Implantology Unit. Faculty of Medicine and Dentistry. University of Santiago de Compostela. Instituto de Investigación Sanitaria de Santiago (IDIS), Santiago de Compostela, Spain; 6 DDs, PhD. Oral Medicine, Oral Surgery and Implantology Unit. Faculty of Medicine and Dentistry. University of Santiago de Compostela. Instituto de Investigación Sanitaria de Santiago (IDIS), Santiago de Compostela, Spain

## Abstract

**Background:**

The main objective of this study is to examine the quality of the information available for patients online with regards to the apicoectomy surgical procedure, both on general and critically selected websites. The hypothesis is that general websites has less quality than other that have been pre-selected.

**Material and Methods:**

A search for the English term "apicoectomy” was performed online. The first 100 websites that appeared in both Google and Yahoo were analysed. Seven validated instruments were used for these two dimensions: quality (DISCERN, JAMA and EQIP), and readability (FRES, Fog Scale, FKRGL and SMOG).

**Results:**

A total of 21 websites (10.5%) were selected. The readability of the websites in both groups was difficult or very difficult. With regards to the quality of the websites, the DISCERN instrument indicated an average value of 2.28 [2.14-2.39] for all of the websites, therefore indicating very low quality with serious defects; however, in the selected websites, the average quality was 3.16 [2.84-3.48], indicating potential, but not serious defects (*p*<0.001). There were statistically significant differences for the FRES values (*p* = 0.030), with a greater readability in the selected group of websites.

**Conclusions:**

We believe that it is very important for the population to become aware of and learn how to use certain exclusion criteria when selecting medical consultation websites, as in doing so, they will be able to obtain a higher quality of information from these websites.

** Key words:**Apicoectomy, readability, DISCERN.

## Introduction

An apicoectomy consists of the surgical removal of the apical portion of a tooth that has a periapical lesion. It forms part of a surgical procedure known as periapical surgery that is performed in order to remove the infectious focus by means of curettage or apical curettage (1-3). In this procedure, the apex is sealed, preventing both filtration and the presence of irritants, therefore ruling out recurrence (4-6).

There are four basic indications for performing the apicoectomy surgical procedure: When the root canal is blocked and it is not possible to perform endodontic retreatment and there are radiographic or clinical signs; in the case of an overextension of the filling material if there are radiographic and clinical signs (7); in the case of endodontic treatment failure if performing endodontic retreatment is not considered to be a suiTable option (acute symptoms, risk of root fracture); and finally in the cases of root perforations with radiographic or clinical signs which are impossible to treat using the orthograde route (8).

As we know, there is a certain degree of complexity involved in the apical surgical procedure and this is why patients must be informed about exactly what the surgical procedure involves (9). Good quality health information is essential in order to ensure greater patient participation in the decision-making processes. The patients and the public require timely, relevant, reliable and easy to understand information. However, communication between health professionals and patients is intrinsically problematic. From the perspective of a health professional, a number of barriers exist, including the use of technical terminology, the volume of information to be conveyed, time constraints, and lack of familiarity with the information on the patient’s part (10-11).

The internet is an international electronic communication network and a mass medium that people can use to look for information on almost any topic, and this includes medical information (12-14). One of the most commonly searched for topics on the internet is information on health problems and health care (15-18). Millions of people around the world use the internet every day to look for information, however, the problem is that these searches are neither precise nor exhaustive (19). Finding information is not the biggest challenge, but rather, the main challenge is finding valid and reliable information. Many factors affect the quality of web-based information. The owners of healthcare websites compete for sales and market participation which can often lead to a selective disclosure of evidence and the inclusion of inaccurate information (20). Another very important factor which must be considered is the fact that the internet is a medium in which anyone with a computer can simultaneously act as the author or editor of content, and in fact, both of these tasks can be carried out anonymously if desired (21-26).

The main objective of this study is to examine the quality of the information that is available for patients online with regards to the apicoectomy surgical procedure. The readability of the websites will be evaluated using the Readability tool. We also intend to study the quality of information, using the following tools: DISCERN, EQIP AND JAMA. In addition, the presence of the HON seal on these websites will be verified. We intend to analyse whether or not the affiliation of the websites affects their quality and readability. Likewise, by using the aforementioned web tools, we aim to determine which search engine is the most suiTable when looking for the specific information which is the subject matter of this study. This will be determined by comparing the results from the website with / without exclusion criteria.

## Material and Methods

- Search strategy

A search for the English term "apicoectomy” was performed online using the two search engines, Google® (www.google.com) and Yahoo!® (www.yahoo.com). The first 100 websites which appeared in both search engines were analysed, in total 200 websites. The websites were listed (10 sites per page. The websites were analyzed independently by two researchers (SAM and MPS). The researchers were calibrated previously, based on a pilot study with 20 websites. The kappa index between both researchers were 0.96. In cases of discordance, a third investigator (AILP) acted as a mediator. The exclusion criteria included: scientific articles, pages that do not work, forums, videos, pages with non-relevant information, medical dictionaries, pages with commercial content and repeated pages. The websites were grouped according to: affiliation (non-profit organisations, for-profit organisations, websites attached to university centres, websites attached to medical centres and websites attached to government entities), specialisation (exclusively related to apicoectomy, or partially related to apicoectomy), type of content (medical searches, clinical trials, of human interest, and questions & answers), and in all cases the presence of the HON (Health On The Net) seal was evaluated. The HON seal recognises websites that provide trustworthy health information and this certification is provided by an independent organisation and examines transparency criteria (including those of the JAMA score) (17), as well as taking into consideration ethical principles, such as whether the website intends to replace, rather than complement the doctor.

- Evaluation procedures: readability and quality 

Seven validated instruments were used to assess the two dimensions: quality (DISCERN, JAMA and EQIP) and readability (Flesch Reading Ease score (FRES), Gunning Fog Index (Fog Scale or GFI), Coleman-Liau Index, Flesch-Kincaid Grade Level (FKRGL), Automated Readability Index and the Simple Measure of Gobbledygook (SMOG).

The readability was evaluated using the automatic tools available on www.readability-score.com, using 4 formulas: FRES, FKRG, GFI, and SMOG index. These were calculated as follows: FRES = 206.835 - (1.015 × average number of words per sentence) - (84.6 × average number of syllables per word), FKRGL = (0.39 × average number of words per sentence) + (11.8 × average number of syllables per word) - 15.59, GFI = 0.4 ((words / sentences) + 100 (complex words / words)), and SMOG = 1.0430 (square root 30 × polysyllables / sentences) + 3.129. Readability grades according to the FRES are 0-30 = very difficult, 30-50 = difficult, 50-60 = fairly difficult, 70-80 = fairly easy, 80-90 = easy, and 90-100 = very easy. A text that is graded as "easy" by the FKRGL is considered as readable by people up to 12 years of age, and a text graded as "difficult" is suiTable for people aged 16 years and over (13). GFI scores are 5 = readable, 10 = hard, 15 = difficult, and 20 = very difficult. The SMOG index outputs to the US school grade level; meaning that the average student in that grade level can read the text [29-30]. The DISCERN instrument consists of 16 items and uses a five-point Likert scale. The first set of items [1-8] deals with the reliability of the publication. A second group of questions [9-15] deals with information on alternative treatments, and a final item considers the overall rating of the content (13,22,24). The quality of information was also assessed using JAMA benchmarks: authorship of medical content (authors and contributors, relevant affiliations, and credentials), attribution (list of references and sources of information), disclosure (website, sponsorship, advertising, commercial financing arrangements, conflicts of interest), and currency (content of the published and updated dates) (17). EQIP was developed as an alternative to DISCERN. It consists of 20 elements that evaluate the following aspects: objective of the document, accuracy and precision of the data, the therapy options and their effect on the quality of life, as well as the advantages, disadvantages and side effects of these therapies. In addition, it contains 7 items that evaluate the language, presentation and design, aspects that are not taken into account when using the DISCERN tool. It uses a 4-level scoring method; if it meets the criterion (1 point), if it partially meets the criterion (0.5 points), and if it does not meet the criterion (0 points). The score is added up to give a maximum of 20 possible points (22).

- Statistical analysis 

The website was considered as the basic unit of analysis. The data was analysed using IBM SPSS Statistics 24.0 software (SPSS Inc., Chicago, IL, USA). The categorical variables were analysed by frequencies. The continuous variables were expressed with average ± standard deviation (SD). The Shapiro-Wilk test was performed in order to analyse the distribution of the variables, and the results revealed the non-normal distribution of the values. Consequently, the Mann-Whitney U test and the Kruskal-Wallis H test were used in a bivariate test in order to search for significant differences between groups, using the search engine (Google® and Yahoo!®), and affiliation (non-profit organisations, for-profit organisations, websites attached to medical centres, websites attached to government entities and websites attached to university centres) as the main variables. Post-hoc tests were performed to establish the differences among the affiliation groups. The value of (*p* <0.05) was established as significant. The Kendall Tau-b test was performed to correlate the accumulated EQIP, DISCERN and FRES and FRGK values. The significance value was set at *p* <0.01.

## Results

The first 100 websites found on Google® and Yahoo!® were evaluated. Out of these, 90 from Google ®, and 88 from Yahoo!® were excluded. A total of 21 websites (10.5%) were selected for analysis, 10 from the Google® search, and 11 from the Yahoo!® search. All of the sites which were included were classified according to affiliation, specialisation and type of content. In Table 1 you can see the descriptive results of the categories that were analysed.

When we analysed the readability of the 21 websites, all of the instruments (FRES, FKRGL, FOG and SMOG) indicated a difficult or very difficult readability, this result only slightly improved when all of the analysed websites were included. With regards to the quality of the websites, the DISCERN instrument indicated a total sum, with an average value of 2.28 [2.14-2.39] for all of the websites, indicating very low quality with serious defects; however, in the selected websites, the average value was 3.16 [2.84-3.48] indicating potential, but not serious defects, that is to say that the selection of pages offered a slightly higher quality.

**Table 1:**
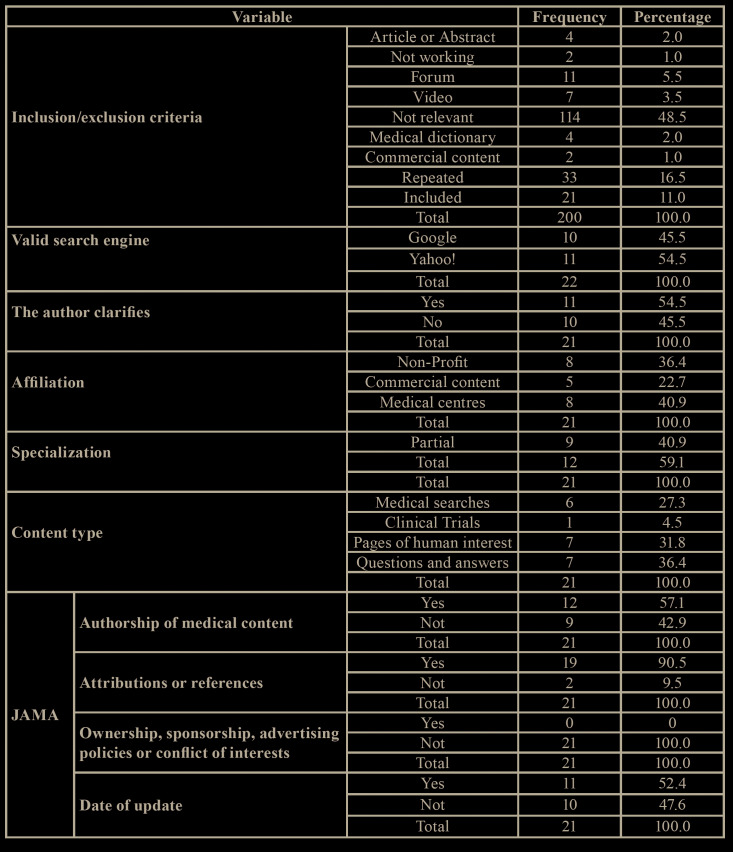
Descriptive results of the indexes and qualitative variables used.

The average values from the JAMA benchmarks were very low in the two groups of websites, less than 1. Using the EQIP tool to evaluate the quality of all of the websites gave a result of 8 [7.72-8.28], and a result of 9.67 [8.93-10.45] for the selected webpages, the score for both groups was less than 10 (the maximum score for this tool is 20). There were statistically significant differences for the FRES values (*p* = 0.030), and a greater readability was observed in the selected group of pages. With regards to the results obtained using the JAMA benchmarks, although the results were bad in both groups, the selected group of websites obtained better results in matters related to authorship and the date of update (*p* <0.001). In terms of quality, both the DISCERN (*p* <0.001) and the EQIP (*p*=0.001) tools offered better results for the group of selected websites.

In a bivariate analysis in which the type of search engine was taken into consideration, we observed some statistically significant differences in the total number of websites using some tools (FRES, FKRGL and DISCERN), however, when selecting the websites, we did not observe differences between the different search engines in terms of the readability or quality (Table 2).

With regards to the analysis stratified by affiliation (Table 3), we observed many statistically significant differences in all of the websites as a whole, however these were reduced once the inclusion criteria had been applied.

**Table 2:**
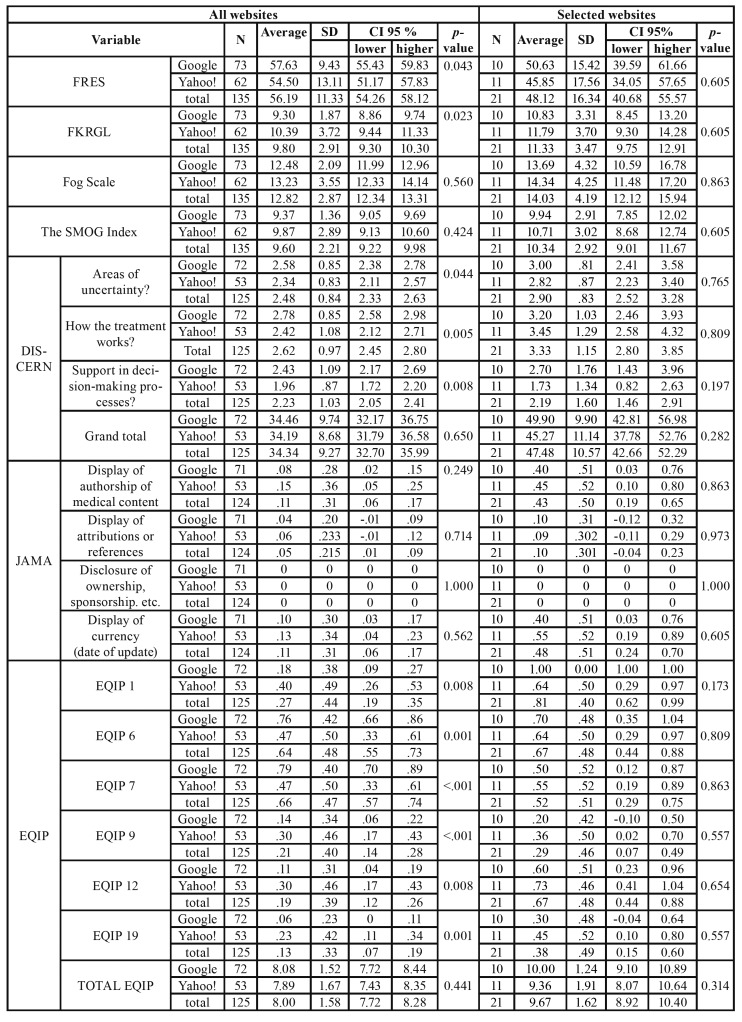
Descriptive data on the statistically significant aspects of the different web tools, depending on the type of search engine, and the group of websites included. SD (standard deviation); CI (confidence interval).

**Table 3:**
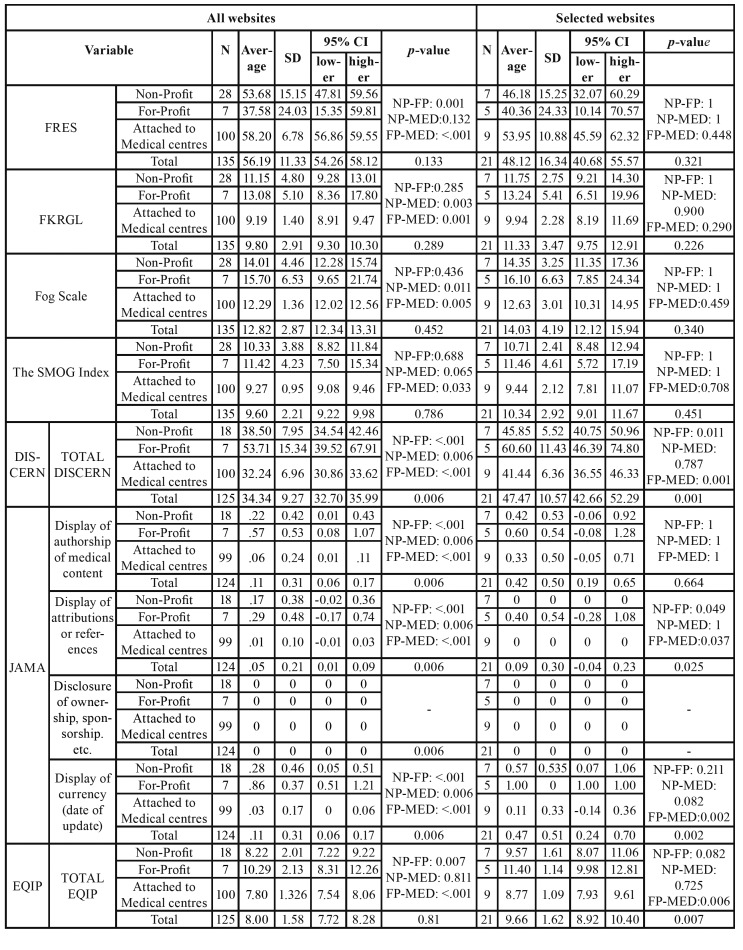
Descriptive data on the different web tools, depending on the type of search engine and the group of websites included. For variables with more than two categories, post-hoc tests were performed to determine the differences between paired groups. NP (Non-profit); FP (Commercial Purposes); MED (Attached to medical centres); SD (Standard Deviation); CI (Confidence Interval).

We observed a better rating for the DISCERN tool in the selected pages for commercial purposes (60.60 [46.39-74.80]) in comparison with the non-profit ones (45.85 [40.75-50.96] and the websites attached to medical centres (41.44 [36.55-46.33]) (*p* = 0.001). With regards to JAMA, differences were observed for matters related to the attribution of references, and the score was also higher for websites with commercial content (*p* = 0.025). With regards to the quality evaluated using the EQIP tool, once again the commercial websites offered the best quality (11.40 [9.98-12.86]) in comparison with the websites attached to medical centres which offered the lowest average values (8.78 [ 7.94-9.62]) (*p* = 0.007).

A high correlation was observed between the readability indexes in both groups, with very high correlation coefficient (CC) values of 0.86-0.92 (*p* <0.001). In terms of quality, the results from the EQIP and DISCERN tools correlated in both groups with a CC = 0.60-0.61 (*p*<0.001). These results verify the validity of the instruments for these websites.

None of the websites presented the HON seal.

## Discussion

The Internet is becoming a predominant source of information in our society. It has revolutionised the way we search for information about medical care. We have seen an increase in patients’ access to health information, and although this may seem positive, the reliability and quality of content found on websites remains a key issue for electronic health consumers given that the information on the Internet is not completely regulated and can potentially mislead the network users. These websites not only need to provide reliable information and accurate content, but the content must also be able to be understood by the target audience. It has also been found that the limited availability of reliable and readable information hinders the patient's ability to make decisions (for example, tobacco cessation) about the various treatment options which are offered to them by the healthcare professionals (27). Most of the websites included in this study (n= 21) had a difficult readability and the content was of moderate-low quality.

We found similar results in readability studies on the information available online about the treatment of leukoplakia, which also had quite a difficult reading level (11). By means of contrast, studies on the information available online about implants, demonstrated that the content of such websites is of an easy reading level (19).

None of the websites presented the HON seal, although this does not necessarily mean that the websites are of poor quality, given that the HON accreditation must be requested and it is not easy for website creators to attain this accreditation. Studies on the information available on leukoplakia confirmed the existence of very few pages with the HON seal (11). In contrast, we observed a greater number of pages on lichen planus with the HON seal (16). We must therefore acknowledge the importance of offering quality and readable information to users, as this will improve communication and trust, and the overall patient-professional relationship.

In addition to this general website analysis, we also analysed the readability and content of the 200 pages according to the search engine used and their affiliation. We observed that the websites from the Yahoo!® search engine were easier to read. On the other hand, by using the DISCERN tool, we were able to observe that the Google® search engine gave us information about areas of uncertainty about the treatment and explanations as to how the treatment works in addition to providing support for decision-making processes. On the contrary, studies into the availability of information on oral cancer observed that Yahoo!® had fewer limitations (28).

The JAMA tool did not demonstrate significant differences between the search engines, which is similar to the results of the studies into the information available on leukoplakia (11).

However, when using the EQIP tool we observed that the websites found by the Yahoo!® search engine were easier to use, as these offered images or diagrams, making it easier to understand the information, likewise, the side effects of the treatment were also indicated. This information is very important for patients as it makes the content more comprehensible. In addition, a greater number of pages in which the authorship was included were offered by this search engine, making these pages more credible (25,26).

In terms of affiliation, we observed that most websites are attached to medical centres, and that said websites contain inferior quality information. However, the websites created for commercial purposes are more complete. These results are in line with those recorded in a study into the information available online on halitosis, in which it was observed that websites created for commercial purposes surpassed the rest (29).

The majority of patients firstly seek information from their trusted professional and if they are unable to provide it, they look for information from other professionals that they find online, therefore we must emphasise the importance of the information that is offered by the dental clinics.

## Conclusions

In this study, by using the tools: DISCERN, EQIP and JAMA, we observed that the information available for patients online about apicoectomy was of a low-moderate quality, and there were also some potential deficiencies, however these were not serious. By using the Readability tool we were able to determine that the content of these pages was difficult to read. We also noted that none of the websites which were analysed presented the HON seal. By analysing the affiliation of these websites, we observed that the highest quality websites were those which had been created for commercial purposes, whilst the lower quality websites were those which are attached to medical centres. When applying certain exclusion criteria to these pages, there were no differences between the results from the different search engines; however, a significant improvement in the readability and quality was noted. We believe that it is very important that the population are aware of, and learn to select medical consultation websites by using certain exclusion criteria, as this will improve the quality of the information that they are able to obtain.
